# The Sanitary Aspect of the Recent Federal Campaign

**Published:** 1863-01

**Authors:** 


### THE SANITARY ASPECT OF THE RECENT FEDERAL CAMPAIGN.

"The secret of the failure of our armies," says an American medical journal of August last, "is comprised in the single word—SICKNESS." "Our present army," writes (two months later) Dr. Edward Jarvis, one of the United States most distinguished statisticians," is in better condition than those of other times and other nations; and more will be done to this end." The first-named authority draws his (as he phrases it) "inevitable conclusion" from the asserted and somewhat cogent facts, if true, that at the moment of writing no less than one hundred regiments were invalided, represented but a fraction of the actual reduction of the Army's physical energy and strength; and that within the short space of three months 50,000 men, it was estimated, had been sent to the rear of the "Grand Army of the Potomac," notwithstanding that during that time it was "within twelve hours" sail of the Capital, and Commissary stores were in unlimited "quantities, at the command of the proper officers," that the army had marched "less than a hundred miles through a rich farming country; and that there was no prevailing epidemic." The last-named authority bases his conclusion chiefly upon a preliminary Report on the Mortality and Sickness of the Volunteer Forces, by the Actuary of the Sanitary Commission, dated May 18th. From this we learn that the annual death-rate of the Volunteer Army, up to that period, was estimated, making allowances for all defects, at 65 per 1000 of the strength. The annual rate of mortality of the British Army throughout the Crimean war was 227 per 1000—the maximum rate being 1173 (January, 1855); the minimum, 72 (June, 1854). In the two months ending the campaign, when the hygienic control of the camps was almost perfect, when provisions were abundant, when the men were well housed and well clothed, when there were no casualties and duty was not excessive, when the death-rate had fallen to the lowest point it reached since the terrible winter of 1854, and when, in short, the sanitary condition of the force would have been well nigh unique in military annals, except from the long-continued fixity of position of the encampments and the serious drawbacks arising therefrom, the mortality did not fall below the annual average of 124 per 1000!

The "constant sickness-rate" of the Federal Volunteer Forces, as given in the Report referred to, is no less remarkable than the death-rate, for it is stated to be but 104 per 1000—that is to say, about double the rate occurring amongst our troops at home under the most favorable circumstances.

If these deductions were to be received unchallenged, well might Dr. Jarvis claim for the United States Volunteers, in the midst of a disastrous war, a supremacy of health-condition above the forces of the chief of European nations in active service. We will not pause to inquire how it comes to pass that forces characterized by such unrivalled health-efficiency (if mortality and sickness are still to be the measure of health) should have proved so unfortunate in the field. Certainly there is no absolute connection between physical vigor and success, notwithstanding that the former in protracted campaigns has invariably been a chief element in securing the latter. It is simply absolute to note that the official returns of sickness and mortality for the Federal volunteers exclude "three months'" volunteers, and "those portions" of the forces which do not make monthly returns to the War Office—facts, by the way, kept somewhat in the background in the official report. The importance of these exclusions will be seen presently.

When, in July, 1861, the camps in the vicinity of Washington were inspected by the Secretary of the Sanitary Commission, he reported that he was "compelled to believe that it is hardly possible to place the Volunteer Army in a good defensive condition against the pestilential influences by which it must soon be surrounded. No general orders (he adds) calculated to strengthen the guard against their approach can be immediately enforced with the necessary vigor. The captains especially have, in general, not the faintest comprehension of their proper responsibility; and if they could be made to understand, they could not be made to perform the part which properly belongs to them in any purely military effort to this end. To somewhat mitigate the result is all the Commission hope to do." On July 27th of the same year, the Sanitary Commission resolved, *apropos* of the discipline of the Volunteer forces,—"That it is the public conviction of the Commission that the soldiers themselves, *in their painful experience of the want of leaders and protectors*, would heartily welcome a rigid discipline exerted over their officers and themselves; that the public would hail with joy the inauguration of a decisive, prompt, and rigid rule, extending alike to officers and men; and that any despondency or doubt connected with our military and national prospects, or with the health and security of our troops, would disappear with the first indications of a rigid order enforced with impartial authority throughout the whole army.

In December, 1861, we are told by the Commission in a report on the hygienic conditions of the Volunteer camp and forces, that "Slovenliness is our (the American) most characteristic national vice." We are told also of the force being inundated by men utterly unfit for service from physical ailments and defects, in consequence of the entire absence of preliminary inspection. The natural result of this state of affairs is seen at the first battle of Bull Run. The Commission informed us that the troops were brought into action badly fed (although close upon their stores), exhausted with fatigue, and, "in respect of discipline, little better than a mob." We have already seen what happened before Richmond with the "Grand Army of the Potomac." "When will our rulers learn wisdom and humanity?" exclaims one of the Inspectors of the Sanitary Commission, after the recent actions in Maryland. "Everything," he says "was wanting that wounded men need, except a place to lie down, and the attentions of personally devoted surgeons (without proper stores, however). The deficiency was greater than usual, for two reasons," he adds: "one, the hurry of the army in passing from a campaign in which everything in the way of supplies was exhausted or lost; the other, the obstruction of the Monocacy, and the want of independent transportation of the (medical) Bureau." Finally, we learn from the Secretary of the Sanitary Commission, in a paper dated the 21st of October, that thirty regiments of one State alone went into the battle of Antietam "absolutely without the smallest particle of medical or surgical stores in the hands of the surgeons;" and that the Government supplies for the relief of the wounded did not reach the ground until the third day after the battle.

What the Sanitary Commission foresaw at the beginning of the war came to pass, as these facts pretty conclusively show, during its course. They are isolated facts; but it needs little experience of military matters to estimate their grave significance in the history of a campaign. They suffice to indicate a condition of things amongst the Federal Volunteers which probably has never yet been witnessed in armies without leading to disaster. We may reasonably conclude that our hebdomadal contemporary across the Atlantic was fully justified in the opinion, that the immediate (whatever might be the remote) cause of the disasters which have befallen the Federal Forces, was SICKNESS. We may further surmise that those portions of the army which did not make monthly returns of sickness and dead were the portions which suffered most from the baneful hygienic conditions to which the men were exposed; and that the exclusion of "three months'" volunteers from the returns which were made, was probably the excision of that portion of the forces—the untried, untrained, and immature—which suffered most from those conditions. —*London Lancet.*

